# Evolution of **β**-Cell Replacement Therapy in Diabetes Mellitus: Islet Cell Transplantation

**DOI:** 10.1155/2011/247959

**Published:** 2011-10-15

**Authors:** Cyrus Jahansouz, Cameron Jahansouz, Sean C. Kumer, Kenneth L. Brayman

**Affiliations:** School of Medicine, University of Virginia, Charlottesville, VA 22102, USA

## Abstract

Diabetes mellitus remains one of the leading causes of morbidity and mortality worldwide. According to the Centers for Disease Control and Prevention, approximately 23.6 million people in the United States are affected. Of these individuals, 5 to 10% have been diagnosed with Type 1 diabetes mellitus (T1DM), an autoimmune disease. Although it often appears in childhood, T1DM may manifest at any age, leading to significant morbidity and decreased quality of life. Since the 1960s, the surgical treatment for diabetes mellitus has evolved to become a viable alternative to insulin administration, beginning with pancreatic transplantation. While islet cell transplantation has emerged as another potential alternative, its role in the treatment of T1DM remains to be solidified as research continues to establish it as a truly viable alternative for achieving insulin independence. In this paper, the historical evolution, procurement, current status, benefits, risks, and ongoing research of islet cell transplantation are explored.

## 1. Introduction

In part one of this two-part paper, pancreas transplantation was explored as the definitive treatment for patients with Type 1 diabetes mellitus (T1DM) [1]. It is estimated that of the 23.6 million people diagnosed with diabetes mellitus, 5–10% consist of patients with T1DM [2]. Moreover, recent reports indicate that the incidence of T1DM is increasing, with one study predicting an increase of 70% in those under the age of 15 by 2020 [3–6]. Accordingly, the significant population already afflicted with this disease compounded by the increasing incidence worldwide will have a tremendous impact on future healthcare both domestically and globally [7]. Estimates show that patients with T1DM treated with intensive medical management have six- to sevenfold higher direct cost than age-matched nondiabetics [8]. Although cost is a concern, it is the long-term complications of T1DM that result in the extensive morbidity in this population which fuel the desire for viable alternative treatments from the standard of care, intensive insulin therapy [9]. Even with the mainstay treatment, patients are still at significant risk for complications including retinopathy, neuropathy, nephropathy, coronary artery disease, peripheral vascular disease, and cerebral vascular disease. While the etiology of this disease remains elusive, it is believed that a relationship exists between genetic susceptibility and environmental factors, including infections and toxins, which results in its fulminant presentation [10, 11].

The quest for a surgical treatment for T1DM first began more than a century ago with the likes of Oskar Minkowski and Josef von Mering at the University of Strasburg, Strasburg, Germany [12, 13]. It was not until 1966 when success was achieved by Kelly et al. who completed the first whole-organ pancreatic transplant at the University of Minnesota [14]. Soon thereafter, the concept of islet cell transplantation, originating with and developed by the visionary Paul Lacy and longtime research partner David Scharp at Washington University in St. Louis, would come into its research phases and be driven further with the likes of John Najarian and David Sutherland at the University of Minnesota [15–17]. Initially, it was met with tremendous optimism. However, the brilliant concept has been troublesome in allowing clinicians to maximize on the idealized potential that lies within it in treating patients with T1DM. Even now, the America Diabetes Association only endorses islet transplantation not as a therapeutic alternative, but rather as “performed only within the setting of controlled research studies” [18].

This paper will now focus on islet cell transplantation as a potentially enhanced alternative therapy for intensive insulin therapy and as a minimally invasive alternative to pancreatic transplantation. It will begin with a brief history of islet cell transplantation, followed by its current state, and then the procedure's benefits and risks. It will continue with a discussion of current research, highlighting barriers and potential therapies, to reduce islet mass loss following transplantation, and imaging as a means to follow the health of the islet mass. It will end with a discussion on islet autotransplantation as it stands today, primarily as an alternative for the treatment of chronic pancreatitis.

## 2. Brief History

Initially, the presence of the exocrine portion of the pancreas proved to be problematic in the transplantation of fragments of pancreas in animals due to the destructive nature of the enzymes [19]. However, this problem was circumvented in 1965 when Moskalewski used collagenase to separate intact islet from a guinea pig's pancreas [19]. Islet cell transplantation subsequently was initiated by Ballinger and Lacy and Reckard et al., who, in 1972, were the first to report that isolated islets could reverse the effects of experimentally induced diabetes [20, 21]. Ballinger and Lacy transplanted 400 to 600 islets obtained from four donor rats intraperitoneally into their diabetic counterparts following the administration of streptozotocin (STZ) to induce the diabetes [20]. After islet cell transplantation, the recipient rats regained their normal weight, reduced their glycosuria, and achieved normoglycemia [20]. A key discovery in islet transplantation was when Kemp et al. compared graft efficacy as a function of graft location. They achieved normoglycemia in STZ-induced diabetic rats through injection of islet cells into the portal vein but not in rats in which islet cells were transplanted intraperitoneally [22]. Monkeys, however, proved to be more challenging. Scharp et al. were only able to partially alleviate STZ-induced diabetes in monkeys, which was attributed to an insufficiency of islet cells as well as allograft rejection [23].

Mirkovitch and Campiche made significant advances when they demonstrated that diabetic dogs could achieve normoglycemia by autotransplantation of pancreatic islet tissue [24]. Using collagenase to digest the pancreas, the partially purified islets were injected into the spleen through the splenic vein [24]. Subsequent splenectomy resulted in a diabetic state [24]. Kretschmer et al. demonstrated that direct injection of the pancreatic tissue into the splenic pulp was more effective than injection through the splenic vessels and the portal vein [25]. Mehigan et al. demonstrated the importance of the size of the minced particles and their influence on the outcome of islet transplantation in dogs. They also observed poor outcomes in relation to acinar cell atrophy and fibrosis from long-term ductal ligation [26, 27]. 

Yet, it was Sutherland et al. in 1974 who began the first human trials to treat diabetes using isolated islets from cadaveric donors [28]. Ten transplants were performed in seven diabetic patients, all of whom had received a prior renal transplant for end-stage diabetic nephropathy. Although a reduction in the exogenous insulin requirement was observed, complete freedom from its use was not achieved. Failure of the grafts could not be attributed to any specific reason but rather secondary to a combination of rejection and inadequate islet cell mass [28]. In 1980, Largiader et al. became the first to report insulin independence following islet allotransplantation in a Type 1 diabetic [29]. The second report was not made until 1990 by Scharp et al. [30]. Socci et al., in a study of six islet-cell transplant recipients with T1DM, also achieved insulin independence in a patient who underwent islet after kidney transplantation. Six months following islet transplantation, the patient achieved insulin independence with normal values of HbA1c, 24-hr metabolic profile, and oral glucose tolerance test. This was sustained for a five-month period [31]. Other subsequent cases were reported throughout the 1990s from the Universities of Alberta, Minnesota, and Pittsburgh [32–34].

Between 1990 and 1995, 180 patients underwent islet cell transplantation worldwide [35]. Of these, 96 were recorded in the international islet transplant registry. 53% of the patients had islet cell function for as long as a week, but graft survival reduced to 26% after one year. Only 7% became insulin independent [35]. In 1994, the University of Giessen introduced protocol changes that significantly improved the efficacy of islet cell transplantation [36]. In all 12 of their patients, the islet graft survived for more than 3 months, and, in 9 patients, the graft functioned for at least a year. Four of these patients attained insulin independence [36, 37]. These results were confirmed independently with significant improvement in graft survival and insulin independence [38, 39].

Throughout the 1990s, and even through today, islet cell transplantation continues to face a number of challenges: transplanting an adequate mass of islets, the adverse effects of the diabetogenic immunosuppression, islet graft loss due to immunologic rejection, identifying an optimal location for transplantation, and overcoming the shortage of pancreata [40–42]. Arguably the most significant advancement in islet transplantation efficacy was made in 2000 by the Edmonton group, whose attempt to address the shortcomings of pancreas transplantation allowed for a tremendous improvement in the islet transplantation protocol. They transplanted an islet mass from two to four donors and avoided glucocorticoids while minimizing the use of calcineurin inhibitors. This was accomplished through use of sirolimus, low-dose tacrolimus, and daclizumab. As a result, they were able to achieve insulin independence in all the seven of their patients but required the use of 15 donor pancreases to do so [43]. In their follow-up international trial, 36 patients with T1DM received 77 islet infusions at nine sites. 16 patients (44%) achieved insulin independence at one year postfinal infusion with 10 patients maintaining partial graft function and the last ten with complete graft loss. These results, thus, confirmed the potential long-term viability and reproducibility of islet cell transplantation, albeit with room for achieving greater results [44].

## 3. Islet Isolation

Some of the most extensive research in islet transplantation has involved identifying avenues for improvement in the steps necessary to isolate an adequate islet cell mass. Following the procurement and preservation of the pancreas, islet cells undergo the following steps: digestion, purification, culture, assessment, and, lastly, transplantation. 

According to CITR, 85% of reported islet transplants employed either the University of Wisconsin (UW) or two-layer methods for pancreas preservation [45]. The two-layer method (TLM), created by Kuroda et al., was developed as a means to increase oxygenation and protect organs from hypoxia through the use of perfluorocarbon during cold preservation [46, 47]. As a result of the oxygenation, adenosine triphosphate production is maintained at the perfluorocarbon and UW interface [47–49].

### 3.1. Digestion

One of the key advances in islet cell transplantation was the development of the automated method of pancreatic digestion with the use of the Ricordi Chamber, based on the work of Moskalewski and Lacy, which was able to increase islet yield [50–52]. Based on CITR, Liberase HI was the most commonly implemented collagenase, used for processing in 77% of cases, followed by Serva Collagenase NB1, used in 18% of cases [45]. Liberase HI was identified in the late 1990s as a collagenase that demonstrated superior enzymatic action over the traditional collagenase preparation (Type P) [53, 54]. However, concerns were raised regarding the small potential risk of bovine spongiform encephalopathy when it was revealed that Liberase HI is isolated from Clostridium histolyticum grown in media containing brain-heart infusion broth [55]. In Japan, three-year follow-up studies of recipients have revealed no incidences of prion diseases [56].

Concerns still remain; thus, research has been conducted for viable collagenase alternatives. Recently, Roche Diagnostics has provided the mammalian tissue-free Liberase MTF-S as an alternative to its Liberase HI [57]. In their study, Shimoda et al. compared four collagenases: Liberase HI, Liberase MTF C/T, Serva Collagenase NB1 Premium Grade, and Clzyme Collagenase HA. They indicated that the three alternative enzymes would enable for higher islet yields than with Liberase HI [58]. When comparing Collagenase XI to Liberase HI, Collagenase XI resulted in a decline in functional capacity of islets which was restored during cultivation. However, Liberase HI exhibited greater functional capacity during isolation and the subsequent seven days of cultivation [59]. O'Gorman et al. compared Liberase MTF to the Serva Collagenase NB1 and observed comparable results between the two collagenases [60]. Of note, in a large-scale comparison of Liberase HI to Collagenase NB1, Liberase was observed to be more efficient for pancreas dissociation but was observed to be more harmful to exocrine cells and islet tissue [61]. Szot et al. at the University of California, San Francisco, have also shown success by implementing the Serva enzyme blend of Collagenase NB1 and Neural Protease NB using a systematic approach and identifying donor criteria to achieve clinically implementable results [62]. Another available collagenase, Vitacyte, was compared with Serva NB1 and not only showed comparable results but exhibited markedly decreased time required to release a significant islet number from acinar tissue, thus, potentially allowing for increased preservation of islet integrity in the future [63]. 

In summary, a number of enzymatic blends and collagenases are available, and more studies are being conducted not only as to their efficacy but also with regards to characterizing the microstructure which defines the pancreas to optimize its dissociation. Consequently, continued research is necessary to identify which product or mixture of components will result in the greatest islet yield and functionality. 

### 3.2. Purification

After enzymatic digestion of the pancreas, the remaining contents then undergo purification to decrease transplanted tissue volume, albeit with minimal loss of islet cells. This step has typically been performed with the cell processor COBE 2991, which uses the differences in density of the islet cells and exocrine tissue to separate them [64–66]. The gradient media implemented in this step have traditionally been the Ficoll solution, first described by Lake et al. in the early 1970s, as another means to improve recovery of islet cells [65]. The process was further advanced by Olack et al., who used the organ preservation solution Euro-Collins to dissolve in which the Ficoll powder [47, 67].

According to CITR, all cases of islet transplantation implemented the use of a density medium [45]. While a number of density media have been researched, Iodixanol has recently revealed positive outcomes in islet yield [68, 69]. Noguchi et al. showed a much larger islet yield when using Iodixanol as compared to Ficoll solutions [68]. This may be attributed to its ability to reduce cytokine/chemokine production, which, as Mita et al. observed, led to a significant reduction in the loss of islet cells during culture [70, 71]. However, this group has observed comparable islet recovery rates to the Ficoll-based density gradient during density purification [70, 71]. 

### 3.3. Culture

As reported to CITR, 54% of islet masses were cultured, defined as six or more hours in a specially prepared nutrient medium, for a median time of 27 hours [45]. Generally, the most commonly used culture medium is the Connaught Medical Research Laboratory-based Miami-defined media no. 1, which has shown positive outcomes compared with alternative media [72, 73]. 

The use of culture has been somewhat controversial when compared with the use of fresh islets and has actually shown a reduction of islet mass and functionality [47, 74–76]. The University of Minnesota recently concluded that, while it is not disadvantageous with regards to recovery of islet function, there is increased expression of several stress-related genes [77]. Nevertheless, culturing islets has some advantages as it allows for functional assessment of islets, preservation during travel time, and recipient preparation in attaining therapeutic levels of immunosuppression [47, 78–80]. Immunologic advantages have also been observed [81, 82]. Furthermore, it may allow time for quality control and for modifications to promote islet survival [47, 80, 83]. 

In a study of 104 islet preparations, Kin et al. identified several factors by univariate analysis contributing to islet loss during culture, including longer cold ischemia time, two-layer method preservation, lower islet purity, and higher islet index. By multivariate analysis, they observed higher islet index and the use of the two-layer method as factors as well. Islet yield also significantly decreased after culture for 20 hours [84].

With regards to modifying the culture to increase islet yield, few potential supplements have emerged. Because of the relative impurity of cultured islets, the presence of exocrine tissue may be problematic in damaging the islet cells. Thus, Loganathan et al. recently showed improved islet recovery while preventing insulin cleavage with the addition of *α*1-antitrypsin (A1AT) to culture, hypothesizing that the added enzyme may protect insulin from cleavage by protease activity [85]. Toso et al. described increased yield with the addition of liraglutide, the long-acting human glucagon-like peptide 1 analogue [86]. 

The temperature at which islets are cultured has also been a point of controversy. Most groups have based their culture at a range of 22–24°C based on the initial work by Lacy et al. [87]. Noguchi et al. recently observed improved outcomes of islet transplantation at 4°C (<5% loss) than that at both 22°C (19% loss) and 37°C (24% loss) [80]. Others have also observed decreased rates of recovery at 37°C relative to the lower temperatures as well [88, 89].

### 3.4. Assessment

While the quantification of islets remains a high priority, assessing their functionality prior to transplantation allows for a predictive component to the procedure to decrease the rate of posttransplant graft failure. The most widely used method is dithizone staining with manual and visual counting of islet equivalents under a light microscope, while viability has been evaluated by assessing membrane integrity with fluorescein diacetate/propidium iodide (FDA/PI) [90]. These methods currently have disadvantages which limit their usefulness. Major limitations include the assessment of three-dimensional islets in two-dimensional planes, lack of ability to identify irreversibly damaged plasma membranes that have not yet permeabilized, operator dependency, its inability to distinguish endocrine (islet) from exocrine (contaminant) tissue, and lack of correlation with mitochondrial function assays, nude mouse bioassay, and clinical outcomes [90]. As such, new methods have been and are currently being developed. 

Computer-assisted digital image analysis has been gaining support as a means of providing more accurate, consistent, and reproducible results in quantifying islet cells. This was recently affirmed by a multicenter study involving all eight member institutions of the National Institutes of Health-supported Islet Cell Resources Consortium [91]. Others have validated this finding as well [92].

Measuring oxygen consumption rate (OCR) and OCR/DNA have gained attention in their ability to predict islet graft function and diabetes reversal [93–96]. Papas et al. applied the measurements in a model for predicting transplant outcome in mice and obtained sensitivity and specificity values of 93% and 94%, respectively. The measurements were also found to be valuable in predicting the marginal mass required for reversing diabetes [97]. Sweet et al. have demonstrated that the glucose-stimulated changes in OCR were predictive of diabetes reversal in mice and that the changes observed were more due to islet cells than nonislet cells [94, 95].

With regards to viability, Papas et al. compared the ratio of ATP to DNA with the ratio of ADP to ATP. They discovered that ATP/DNA as a better measure of viability as ATP levels fluctuate significantly and reversibly with metabolic stress [96]. They also cited the substantial disagreement that exists as to the significance of the ADP/ATP as well as a reason for its limited use [98–100].

The University of Wisconsin recently presented a multiparametric objective approach to assess islet quality based on mitochondrial membrane potential (MMP), in vitro glucose-stimulated insulin secretion (GSIS), and ATP to ADP ratio as a marker of reduced oxidative phosphorylation and achieved an accuracy of more than 86% in predicting in vivo functional potency [101]. 

## 4. Current State and Statistics

Of the 46 islet transplant centers polled in North America by the Collaborative Islet Transplant Registry (CITR) from 1999 through 2008, 32 centers performed at least one islet allograft transplant, with 27 of those centers reporting detailed information to the registry [45]. From that time period, the CITR report includes 81% of both human islet allograft recipients and procedures conducted in North America. It includes 412 recipients of islet transplants receiving 828 infusions from 905 donors. Of the 412 recipients, 347 (84%) received islet-alone (IA) infusions while 65 recipients (16%) had received a kidney transplant prior to receiving islet infusions (IAK: Islet After Kidney). Both mean recipient and donor age was 44 years, with recipients characterized by a mean duration of diabetes of 28 years. Mean time from cross-clamp to pancreas recovery was 44 minutes, while cold ischemic time was 7.3 hours. Only 11–15% of patients remained insulin-independent throughout the first year [45].

**Figure 1:**
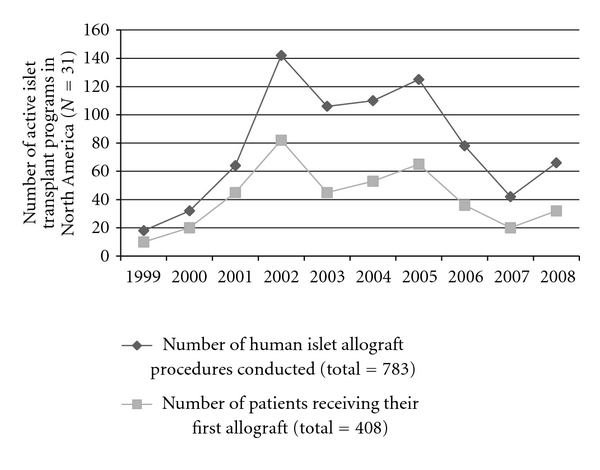
Total number of islet transplant recipients and total infusions in North America, 1999–2008, based on the CITR 2009 Annual Report [45].

**Figure 2:**
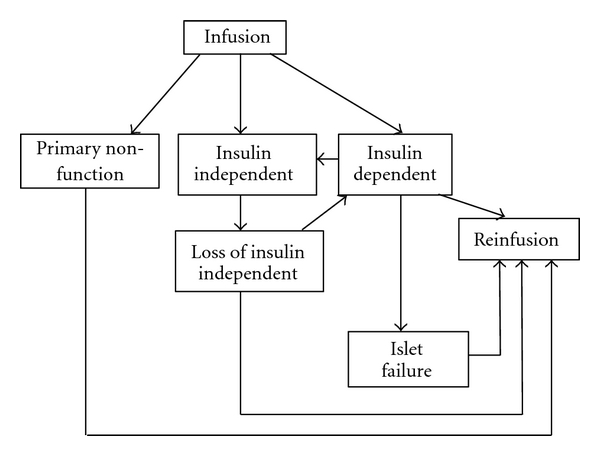
Possible states after first infusion.

**Figure 3:**
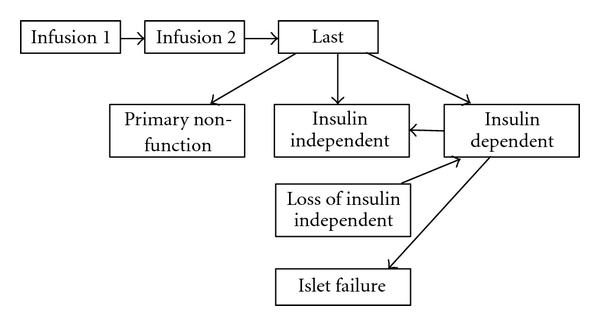
Possible states after last infusion.

Figure 1 illustrates the total number of islet transplant procedure performed and the number of recipients in the 32 active North American transplant centers [45]. Figures 2 and 3 illustrate possible states after first and last infusion [45]. In 2008, 66 islet allograft procedures were performed, with 32 patients receiving their first allograft. Both are an increase from 2007, in which 42 procedures were performed with 20 patients receiving their first allograft. However, these are still only approximately half the number of procedures performed and patients receiving a first allograft compared to 2005 [45, 102].

By the end of the first year following islet infusion, 65% of IA patients were reinfused. 8–12% of IA recipients retain detectable C-peptide while being insulin dependent. Without reinfusion as a factor, insulin independence in IA recipients declines to 27% at year 3. Furthermore, from the last infusion, the rate of loss of islet function steadily increases from 12% at month 6 to 42% at year 4. The proportion of patients retaining graft function with exogenous insulin over the three-year period remains in the range of 19–31%. Similar to IA recipients, postfirst infusion rates for IAK remain near 20%. Postlast infusion rates remain consistently below those of IA recipients through the four-year period as well. These trends of increasing graft loss and decreasing insulin independence over time following infusion prevail regardless of the total number of infusions given, although these rates differ somewhat [45].

If the number of infusions is taken into consideration, a second- or third-repeat infusion has a more significant role in increasing the proportion of insulin-independent recipients from the beginning of infusion to the 500-day period after infusion. Thus, the greater number of infusions a patient receives, the quicker the recipient will attain insulin independence. Thereafter, the percentage of insulin-independent patients declines at a similar rate until the 900 day mark to the 1100-day mark (3-year mark) regardless of the number of infusions. Of the patients who attain insulin independence, 70% maintain this status after one year, and 45% maintain it at three years. Furthermore, graft function continues to decrease over time as well, with 35% of all recipients losing graft function at the three-year period after their last infusion [45].

At this point, it may be logical to compare the results of islet cell transplantation that of pancreas transplantation. However, a true comparison with regards to graft function at this time cannot be performed due to the far superior results of pancreas transplantation. Following pancreas transplantation, 1-year posttransplant graft survival remains ~78–85% and at 3 years, 60–80%. Patient survival in pancreas transplantation at 1 year exceeds 95% for all three categories: Simultaneous Pancreas Kidney (SPK), Pancreas After Kidney (PAK), and Pancreas Transplant Alone (PTA) [103–105]. 3-year survival rates exceed 90%. Speight et al. did perform a review of twelve studies which compared patient-reported outcomes (PRO) of Pancreas After Kidney (PAK), Pancreas Transplant Alone (PTA), IAK, and IA transplantations, and found benefits with regards to fear of hypoglycemia, diabetes-specific quality of life, and general health status. On the other hand, shortcomings were observed with short-term pain, immunosuppressant side effects, and depressed mood associated with loss of graft function. Thus, as they concluded, much has yet to be learned in terms of patient-based quality-of-life outcomes in comparing the different types of pancreas transplantation with islet cell transplantation [106]. 

Optimistically; however, while there is no standard tool to effectively monitor islet cell rejection, Toso et al. monitored the immune reactivity against islet cell grafts in mice using enzyme-linked immunosorbent spot (ELISPOT) assay to identify the ex vivo release of *γ*IFN from splenocytes stimulated by islet donor extracts. They were able to demonstrate transiently increased levels of immune reactivity, as indicated by reactivity of splenocytes against islet proteins, in allogeneic models, and were able to achieve a sensitivity of 70% and specificity of 94% [107]. In the future, such data, combined with the gradual improvement in islet efficacy, may prove to influence and help guide the patient's decision as to the appropriate treatment thereafter.

## 5. Benefits

Islet cell transplantation has been endorsed as having a largely beneficial impact by several groups with regards to achieving stronger metabolic control over brittle diabetes and reducing the tremendous physiologic impact of T1DM. Although improvements in the counterregulation and symptom-recognition mechanisms with respect to glucagon and epinephrine may be observed, values continue to be considerably below normal [108–110]. However, this drawback may be mitigated as growth hormone levels are restored and normalized [107]. Furthermore, autonomic and neuroglucopenic hypoglycemia warning symptoms return even in individuals with longstanding diabetes [111]. Long-term benefits of islet graft function include near-normal HgbA_1c_ levels and reasonable glucose control with occasional insulin independence [112]. According to CITR, the percentage of IA recipients with normal HbA_1c_ levels increased from 2% preinfusion to 51–60% at year one after last infusion [45]. In a study of seven IAK transplant recipients, a near two-point reduction in HgbA_1c_ was observed with 30% achieving 1 year insulin independence and 86% with one year graft function. No severe hypoglycemic events were reported [113]. In their study, Warnock et al. enrolled 10 patients with diabetes-induced renal dysfunction in a best medical therapy program and then crossed them over to islet transplantation. All patients showed improved metabolic control reducing HbA_1c_ from a mean of 6.9% after best care to 6.2% 6 months after islet transplantation [114]. Poggioli et al. observed significant nutritional and dietary changes in 30 of 52 islet transplant recipients, including substantial reductions in body weight, body mass index, waist circumference, and fat weight [115]. Considerable progression of diabetic retinopathy is also much more likely in patients with intensive insulin therapy as opposed to islet transplant recipients, in whom it was shown to stabilize [116, 117]. 

Cardiovascular function improved as well in patients with end-stage renal disease (ESRD) receiving both kidney and islet transplants relative to patients receiving kidney only, with improvements in atherothrombotic profile and endothelial morphology [118]. The same IAK group also had improvements in ejection fraction and peak end-diastolic volume (EDV) and stabilization in time to peak filling rate. These indices were diminished in the kidney-only group [119]. Furthermore, renal graft survival and function were also improved when combined with islet cell transplantation [120]. Poor long-term outcomes of polyneuropathy were also prevented in patients undergoing the IAK procedure, as evident with a reduction in advanced glycation end products (AGEs) and expression of their specific receptors (RAGE) [121]. Lee et al. have also shown that patients may stabilize or even demonstrate improvement of their diabetic neuropathy [117].

## 6. Risks

In the short term, the risk associated with islet transplantation is pointedly less, in comparison with whole-organ pancreatic transplantation. However, similar to pancreas transplantation, longer-term complications are likely associated with the chronic necessity for immunosuppression and are highlighted by the well-known calcineurin inhibitor-induced nephrotoxicity, which becomes more important due to the potential preexistence of diabetic nephropathy [122–125]. 

The Edmonton Group, in a review of 34 patients undergoing 68 procedures, recorded potentially serious complications in only 6 of 68 procedures [126]. Complications included two patients with portal venous thrombosis and four patients with clinically significant intra-abdominal hemorrhage [126]. Bleeding was also observed in 18 of 132 percutaneous transhepatic islet transplants in 67 patients by Villiger et al. from 1999 to 2005. However, they did conclude that the complication is avoidable if the intraparenchymal liver tract is sealed effectively [127]. Maleux et al. reported on 15 patients who underwent 31 procedures. Only three patients presented with complaints of transient abdominal pain, which furthered the notion that percutaneous transhepatic injection of islet cell grafts is a safe and reproducible procedure [128]. From 1992 through 2003 at the University of Geneva Hospital in Switzerland, 62 percutaneous transhepatic injections were performed. Nine complications (14.5%) were observed, of which two were portal vein thrombosis and seven were intra-abdominal hemorrhage [129]. In their study of seven IAK recipients, Cure et al. reported two procedure-related pleural effusions and one episode of cholecystitis, all of which resolved [113].

As mentioned above, sensitization is another potential threat following a failed islet transplant. This was illustrated by the Edmonton Group in which 16% of the recipients became sensitized after transplantation, with *de novo* antibodies seen in 36% of sensitized and 33% of nonsensitized recipients [130]. In their international trial, they reported procedural-related complications including acute intraperitoneal bleeding in 7 of 77 (9%) with four requiring blood transfusions and the other requiring laparotomy. No cases of portal vein thrombosis were reported. Two of the 36 patients had partial portal branch vein occlusions, but they were successfully treated with anticoagulation [44].

## 7. Ongoing Debate and Limitations

Central to the debate has been whether insulin independence should be the main objective in islet cell transplantation or whether it should simply be to achieve acute and long-term metabolic control and to improve the quality of life of individuals with brittle diabetes [131]. At the present time, support lends itself to the latter. Once again, the international trial of the Edmonton protocol concluded that, even with normal endocrine reserves rarely being achieved, insulin independence is gradually diminished over time. Considerable metabolic control, however, is achieved with protection from hypoglycemia and improved HgbA_1c_ levels, thus, favoring the procedure for highly selected patients after exhausting all other therapeutic options [44]. Recently, the GRAGIL group released similar results favoring the use of islet transplantation as a therapeutic means to achieve stronger metabolic control with respect to restoring beta-cell function rather than measuring success by the achievement of insulin independence [132].

One observed limitation of islet cell transplantation is the great variation in achieving insulin independence from center to center worldwide, which is primarily attributed to a lack of experience [43]. Another study by the GRAGIL Consortium proposed a solution to bypass this problem by being the first to employ the Edmonton protocol in a multicenter setting [133]. They have demonstrated further feasibility of the multicenter approach by illustrating the absence of ill effects with regards to shipment of islet cells, discussed below [134]. 

To combat the limitations associated with islet cell transplantation, it may be best to steer the therapy towards those patients with the most potential for graft survival and who may benefit the most: high-risk patients with recurrent episodes of hypoglycemia [135]. Two of the most important and recurrently identified aspects regulating islet survival have been auto- and alloimmunity and the maintenance of a sufficient islet cell mass. At the Leiden University, it was shown that the presence and amount of autoimmunity to one or two antigens determine the survival of islet grafts and, as such, imply a role in patient selection in the future to maximize graft efficacy and adjust graft size as needed [136]. Ironically, it has recently been shown that the immunosuppression regimen implemented by the Edmonton protocol may exacerbate this autoimmunity. Monti et al. recently reported that the protocol may actually be causative in the long-term failure of islet cell transplantation. Employing the protocol often results in lymphopenia that is associated with elevated serum levels of the homeostatic cytokines IL-7 and IL-15, which expands the autoreactive CD8^+^ T-cell population [137].

### 7.1. Problems with Immunosuppression

As has been documented, many of the immunosuppressants required in islet transplantation have also been shown to adversely affect the transplanted islets. One such familiar consequence of the use of corticosteroids is hyperglycemia as a result of insulin resistance occurring from the reduction of insulin-mediated glucose uptake and utilization [138]. Both Sirolimus and Tacrolimus inhibit beta-cell regeneration and prevent the normalization of glucose homeostasis in treating diabetic mice as well [139]. Tacrolimus has also been observed to decrease insulin gene transcription, the stability of insulin mRNA, in vitro insulin synthesis and mitochondrial density, and in vivo insulin secretion, while Sirolimus decreases in vitro insulin synthesis and secretion, ductal cell regeneration, and angiogenesis [140–149]. Additionally, mycophenolate mofetil is a potent inhibitor of ductal neogenesis and has been shown to impair glucose-stimulated insulin secretion [150, 151]. However, Johnson et al. have shown that, to some extent, these negative effects may be counteracted with the glucagon-like peptide-1 exenatide. The use of exenatide has shown positive effects on the islet cell graft in stimulating insulin secretion and improving graft function, thus, aiding in glycemic control [151–157].

While a variety of groups have been able to achieve insulin independence with single donor islet transplantations, the protocol for this achievement still varies from group to group [158]. The University of Minnesota achieved insulin independence in all the eight of its patients with each patient receiving only one islet graft. Their protocol consisted of daclizumab, etanercept, and thymoglobulin for induction, with mycophenolate mofetil, Sirolimus, and either no or low-dose Tacrolimus. Five of eight patients maintained insulin independence beyond one year, and, in the three patients who experienced graft failure, it was preceded by subtherapeutic Sirolimus exposure without measurable Tacrolimus trough levels [78]. Improved longer-term outcomes have been achieved at the University of Minnesota as well. Six patients underwent one or two islet graft infusions with a protocol of thymoglobulin for induction along with etanercept, cyclosporine, and everolimus for maintenance for the first year following transplantation. Thereafter, mycophenolate mofetil or mycophenolic acid substituted for everolimus. Five patients were insulin independent at one year, while four remained so at 3.4 +/− 0.4 years after transplant [159].

At the Emory University, they compared the Edmonton protocol, highlighted earlier, with a protocol consisting of daclizumab induction, a 6-month course of Tacrolimus, and maintenance with efalizumab and mycophenolate mofetil. While two patients achieved insulin independence in the Edmonton protocol, all four paitnets with the novel protocol did so [160]. In another study, Matsumoto et al. compared two common immunosuppression protocols on six patients: in the first, three patients were placed on daclizumab for induction, with Sirolimus and Tacrolimus for maintenance along with etanercept as an anti-inflammatory agent; while in the other, three patients were not only placed on thymoglobulin for induction and tacrolimus and mycophenolate mofetil for maintenance along with anakinra (anti-IL-*β*) and etanercept but also provided islet cells with iodixanol purification. While all patients became insulin independent, the former protocol required two infusions to do so [161].

At the University of California, San Francisco, a group of ten patients with T1DM underwent islet transplantation and was treated with a protocol of thymoglobulin induction and maintenance with Sirolimus or mycophenolate and either belatacept (BELA) or efalizumab (EFA). While EFA is no longer available for clinical use, all five patients who received BELA achieved insulin independence after a single islet graft, with only one requiring insulin use 305 days following transplantation [162].

With increasing knowledge of the negative effects of certain immunosuppressants on *β*-cell function, it is hopeful that novel protocols will continue to develop, and ones that have achieved success in smaller populations will be implemented on a larger scale so that standardized protocols may be established. Thus, one may be optimistic that improved protocols may lead to stronger results in the near future.

### 7.2. Inflammation, the Immune Response, and Oxidative Stress

Perhaps the major barrier in islet transplantation is the inevitable decline of islet graft function over the short and the long terms. Shortly after intraportal transplantation, more than 60% of islet cells undergo apoptosis during the revascularization period [163–165]. During the engraftment process, which may last up to two weeks, oxygen is received primarily through passive diffusion, thus, creating an environment of oxidative stress [166, 167]. This hypoxic state is an important contributor to islet dysfunction with resultant apoptosis and necrosis [168, 169]. One of many facets of this hypoxic injury is the role that inducible nitric oxide synthase (iNOS)-nitric oxide (NO) has in signaling apoptosis [170, 171]. Another is the hypoxia-induced activation of AMP-activated protein kinase in cytokine-induced apoptosis [172–174]. Subsequently, increased metabolic demand is required of the remaining islets, which may lead to metabolic exhaustion and dysfunction [175].

Defenses, consisting of the innate and adaptive immune responses, also contribute to the substantial islet cell loss [176]. The innate immune system creates an environment ill suited for the survival of the sensitive islet cells. Conversely, the adaptive immune response is better controlled with current immunosuppressive protocols [176, 177]. Not surprisingly, cytokines and low-grade systemic inflammation promote islet cell dysfunction and death as well [178–181]. One such cytokine is Tumor Necrosis Factor (TNF)*α* and its known toxicity to islet cells [180, 181]. Thus, as noted above, the implementation of etanercept has been more widely implemented in recent studies at multiple institutions with promising results [78, 159, 161]. It is hopeful that these results will be observed once again in the multicenter Phase 3 trial implementing the Clinical Islet Transplantation Protocol 07 currently being conducted by the Clinical Islet Transplantation Consortium [182]. The inflammatory environment in which islets are placed continue to form a central barrier to successful graft survival, and targeting it at different levels may achieve more successful results [177].

A number of studies have shown that an avenue of improving islet viability may lie with the role that Toll-like receptor (TLR) activation has in mediating early islet graft failure. As part of innate immunity, it activates pathways such as NFkB, and, if it is inhibited or even partially suppressed, it may aid in the grafting process [183–185]. Goldberg et al. have shown that carbon monoxide exposure to isolated donor islets may in fact provide some protection by blocking the TLR upregulation that occurs during the isolation procedure [186]. Following activation of the transcription factor NFkB pathway, there is upregulation of genes mediating inflammation and apoptosis, thus, supporting its role as one of the mechanisms of islet loss as well as its blockade as a potential therapy [187–192]. An additional potential target may lie with the high-mobility group box 1 (HMGB1) due to its role in mediating early graft loss by stimulating hepatic mononuclear cells, upregulating CD40 expression, and enhancing IL-12 production by dendritic cells [184, 193].

An alternative avenue of immunologic research lies within the role of chemokines, notably monocyte chemoattractant protein-1 (MCP-1)/CCL2, constitutively expressed in islet cells and their role in monocyte recruitment, insulitis, islet engraftment, and graft destruction [194–198]. Ogliari et al. have shown that higher donor levels of MCP-1/CCL2, as seen with brain death, lead to decreased graft survival in SPK recipients, likely further contributing to the posttransplant inflammatory state [199]. Similarly, Saito et al. observed a high expression of both tissue factor and MCP-1/CCL2 expression in isolated islets resulting from brain death and ischemic stress in the rodent model, thus, emphasizing a role for pancreatic management from brain-dead donors [200]. Melzi et al. have suggested that strategies to decrease recipient MCP-1/CCL2 may be more fruitful [201]. Lee et al. indicated beneficial results in the mouse model when blocking MCP-1/CCL2 binding to its receptor, CCR2 [202]. Interestingly, two key mediators in the chemokine's release are NFkB and Angiotensin II, which is actively generated in the pancreas, through their increased expression of MCP-1/CCL2 mRNA and protein [203, 204].

Correspondingly, a few adjunctive therapies have shown potential in improving islet survival. A role may exist for adenosine A(2A) agonists as they improve glucose-stimulated insulin secretion and inhibit inflammatory islet damage in the peritransplant period [205, 206]. Intensive insulin and heparin administration have also shown benefit in the peritransplant period [207]. Heparin's beneficial effects likely stem from its favorable impact against the instant blood-mediated inflammatory reaction (IMBIR) [83, 207]. 

While research continues to illustrate the barriers that exist in the peritransplant period, several potential therapeutic targets have been characterized, with a few therapies showing benefit. Work still remains in this phase and will only continue to shed light on the tremendous immunological underworks that characterize this crucial time frame of islet stress.

### 7.3. Optimal Location

A critical facet of islet cell transplantation remains the optimal site of implantation. As noted above, Kemp et al. were the first to demonstrate success with intrahepatic transplantation and thus has remained as a favored site for some time [22]. Glucagon unresponsiveness to hypoglycemia remains a consideration, as noted earlier, which is in contrast to that seen in whole-organ pancreatic transplantation. This is thought to be due to the increased intrahepatic glucose flux masking systemic hypoglycemia [208–213]. Liver ischemia and procedure-related complications, such as hemorrhage and thrombosis, are also concerns [214–217]. 

Accordingly, several locations have been considered as possibilities for the future, including vascular (celiac artery, spleen, lung), organ (renal subcapsule, pancreas, intramuscular, omental pouch, intraperitoneal, subcutaneous), and immunoprivileged (intracisterna magna, testis, and thymus) sites [217]. Recently, Kim et al. compared the kidney, liver, muscle, and omentum as islet transplant sites, evaluating each based on operative feasibility, implantation efficiency assessed as marginal mass required and mean time to achieve normoglycemia, and glycemic control in the mouse model [218]. They observed that the omentum may be an optimum site in terms of implantation and efficiency, albeit with disadvantages. Namely, it does not allow repeat transplantation, and it is not possible in patients with a past laparotomy. On the other hand, muscle offers ease of operative feasibility but less vascularity. While the liver resulted in much greater mortality and delayed graft function, it afforded greater marginal mass. Interestingly, the kidney produced excellent results in feasibility, efficiency, and glycemic control, but, as noted, differences exist with respect to the human kidney, as the subcapsule allows for less elasticity and affords limited space [218, 219]. Recently, the femur bone marrow cavity has also been introduced as a potential site of transplantation of a bioartificial pancreas (BAP), as reported by Yang et al. The BAP was composed of mouse insulinoma cells encapsulated in agarose gel further enclosed in a calcium phosphate chamber [220]. The group also evaluated the possibility of applying the BAP to intramuscular space in a comparison with the intramedullary cavity but reported increased effectiveness with the latter [221].

Further research has recently been performed with regards to an intramuscular transplant site. Directly comparing the intraportal site to muscle (biceps femoris) in rats, Lund et al. observed twice the necessary IEQ to achieve normoglycemia in muscle [222]. Others have shown some feasibility of intramuscular implantation as well, observing much better oxygenation when compared to the renal subcapsular site in rats but naturally less oxygenation than that in native pancreatic islets of nontransplanted controls [223]. Of note, Christoffersson et al. showed the importance of neutrophils in restoring intraislet perfusion following transplantation at an intramuscular site [224, 225]. 

Performed in a variety of ways, several reports have been published with regards to increasing the vascularization of islet cells transplanted intramuscularly or subcutaneously. As will be discussed later, Witkowski et al. achieved excellent results when pretreating intramuscular sites with a biocompatible angiogenic scaffold before transplantation [226]. Salvay et al. created microporous polymer scaffolds produced from copolymers of lactide and glycolide, which were then adsorbed with collagen IV, fibronectin, laminin-332, or serum proteins before being seeded with 125 mouse islets. The scaffolds were then implanted onto the epididymal fat pad in mice. The scaffold with collagen IV maximally enhanced graft function promoting graft efficacy [227].

A number of studies have shown the potential clinical impact adjunctive treatment with VEGF has on increasing islet graft efficacy and viability [228–232]. Stiegler et al. used a combination of foam dressing, vacuum-assisted wound closure, and hyperbaric oxygenation (HBO) in rats, with results indicating increased vessel ingrowth and vascular endothelial growth factor (VEGF) levels dependent on duration of HBO treatment. Perfusion was significantly improved in the experimental group with only a small amount of apoptosis following transplantation [233]. Similarly, islet cells transplanted subcutaneously with adipose tissue-derived stromal cells (ADSCs), and minced adipose tissue showed increased vascularization and higher capillary density than mice implanted with either ADSCs or minced adipose tissue alone [234]. Ohmura et al. indicated that ADSCs promote survival and insulin function of the graft and reduced the islet mass required for reversal of diabetes [235]. Ito et al. demonstrated improved islet graft function and promotion of graft revascularization when islet cells were cotransplanted with bone marrow-derived mesenchymal stem cells in rats [236]. Duprez et al. were able to create composite cells of mesenchymal stem cells and islet cells, and they showed beneficial results with regards to minimizing the immune reaction with blood and suppressing lymphocyte proliferation [237]. Other groups have observed similar immunosuppressive results and improvements in vascularization with mesenchymal stem cells as well [238, 239]. 

In another novel study, Shimoda et al. used an ultrasound-mediated gene-transfer method named ultrasound-targeted microbubble destruction (UTMD) to deliver nonviral plasmid vectors encoding VEGF into the host liver of mice. They observed that the *VEGF* gene promoted islet revascularization following transplantation and improved rates of achieving normoglycemia [240]. Similarly, Kheradmand et al. created an innovative approach to transplant islets through a combination of mechanisms. They created an extrahepatic site by transplanting islet-loaded microporous poly(lactide-co-glycolide) (PLG) scaffolds into the epididymal fat pad in mice. Ethylcarbodiimide- (ECDI-) treated splenocytes were infused as a tolerance induction strategy. Altogether, they experienced excellent results superior to intraportal transplanted islets [241]. Vaithilingam et al. observed increased levels of hypoxia-inducible factor-1*α* (HIF-1*α*) and VEGF expression when transplanting encapsulated human islets pretreated with desferrioxamine (DFO) into the peritoneal cavity of mice [242]. Others have noted the use of DFO to stimulate VEGF expression and islet vascularization as well [243–245].

While the intrahepatic site is the classic location for islet transplantation, it is likely that other sites will take over this role. A number of novel approaches have been discussed, and, considering the improvements observed with inducing vascularization, an intramuscular location may prevail as the leading candidate to replace the liver.

### 7.4. Shortage of Supply

Another major hindrance to human islet cell research and transplantation remains the shortage of pancreata. Potential solutions to increase resources lie in stem cells and xenotransplantation, both of which are being extensively researched, and in international islet shipping. At this point, we will focus on recent studies involving the shipment of islet cells. Vaithilingam et al. recently demonstrated success of shipping encapsulated islets from Chicago, Ill, to Sydney, Australia, achieving a recovery rate of 88%. Islets were encapsulated with a barium alginate microcapsule and were isolated for a median total of 11 days before being transplanted in mice [246]. Similarly, Qi et al. showed success with long-distance shipping of encapsulated alginate calcium/barium microbeads, maintaining in vitro and in vivo islet function [247]. Other groups have established the improved survival and functionality of alginate-encapsulated islets as well [248–250]. Ikemoto et al. have also had some success, shipping islets from Dallas, Tex, to Fukuoka, Japan. Islets were packed in either gas-permeable bags or non-gas-permeable bags. Recovery rate was higher in the gas-permeable group than the nongas-permeable group: 56.4 ± 10.1% versus 20.5 ± 20.6%, *P* < 0.01. Purity also decreased to a greater extent in the nongas-permeable group [251]. Ichii et al. have endorsed the use of gas-permeable bags as well and have promoted shipments following cultured islets as opposed to that immediately after isolation [252]. Because of the changes in pressure and temperature islets must endure during shipment, Rozak et al. have suggested the use of containers equipped with commercially available TCP Phase 22 phase change material (TCP) and custom-designed pressure regulated gyroscopic shipping containers (PRGSC), which illustrated excellent environmental control by limiting temperature and pressure changes [253]. 

## 8. Further Research

Optimistic findings with regards to genetic manipulation have been observed, with caspase inhibition showing promise. Islet cells transduced with an X-linked inhibitor of the apoptosis protein (XIAP) expressing recombinant adenovirus were resistant to apoptosis. By inhibiting caspases 3, 7, and 9, this reduced the required transplanted islet cell mass [254–256]. However, a drawback remains the required use of adenoviral gene therapy. 

Expanding on caspase inhibition, Emamaullee et al. employed a short course of the caspase inhibitor zVAD-FMK and demonstrated efficacy in enhancing marginal mass posttransplant grafting. Consequently, this illustrated the extent of damage caused to the islet implants by ischemia. zVAD-FMK selectively inhibits caspases 1–10 and 12. With renal subcapsular islet infusion, 90% of zVAD-FMK-treated mice became euglycemic with 250 islets versus 27% of the control animals. With portal infusion, 75% of zVAD-FMK-treated animals established euglycemia with only 500 islets, and all of the controls remained severely diabetic. No systemic toxicity was demonstrated [257].

In another study, Emamaullee et al. utilized another caspase inhibitor, EP1013 (zVD-FMK), which selectively inhibits caspases 1, 3, 6, 7, 8, and 9, as opposed to the less specific zVAD-FMK. No discernable difference was observed between the two caspase inhibitors with islets injected in the subcapsular space, but there was a significant difference observed with islets transplanted intraportally. Nearly 100% of the EP1013-treated animals achieved euglycemia with 500 islets, while only 62.5% of zVAD-treated animals, and 0% of the controls established euglycemia. Once again, no systemic toxicity was observed [258]. 

More recently, the group conducted another study to observe the combined effects of EP1013 with CTLA4-Ig, a costimulatory blocking agent shown to be an effective immunomodulatory agent [259]. Fully major histocompatibility complex (MHC) mismatched mice underwent islet allotransplantation. 40% of mice which were administered CTLA4-Ig alone resulted in prolonged islet survival of greater than 180 days, whereas 91% of mice administered both EP1013 and CTLA4-Ig showed prolonged survival. Treatment with EP1013 alone did not result in prolongation of allograft survival. Furthermore, in the study, they showed that the complimentary effects of both drugs reduced the frequency of intragraft CD4+ and CD8+ T cells both at short and long terms, and reduced the functional alloreactive T cell response along with B-cell allosensitization [260]. Thus, there is reason for optimism that this type of therapy could dramatically reduce the number of islets required to induce insulin independence, reduce early immune stimulation from dying islets, and improve current immunosuppressive regiments to decrease or rid of the need for nephrotoxic agents. 

At the University of Pittsburgh, growth factors, such as hepatocyte growth factor (HGF), and signaling molecules, such as protein kinase B (PKB)/Akt, have also shown promise [261]. Furthermore, combination gene therapy may have a role in posttransplant therapy, as shown by the co-expression of VEGF and interleukin-1 receptor antagonist and its resultant success in islet survival [262].

## 9. Imaging

Central to monitoring the progression of islet cells following transplantation is the role noninvasive imaging will have in the future, and, to this end, magnetic resonance imaging (MRI) is one of the major imaging modalities which may prove valuable. In 2004, Jirák et al. was the first to report a technique for in vitro labeling of isolated pancreatic islets with the MR-contrast agent Ferucarbotran, composed of crystalline iron nanoparticles with superparamagnetic properties coated by carboxydextran, allowing for increased hydrophilicity and increased uptake by cells [263, 264]. Ferucarbotran is uptaken by islet cells by means of endocytosis without subsequent deleterious effects on function [265–267]. The feasibility and safety of this model, specifically iron labeling, to humans was then demonstrated by Toso et al. in four patients receiving a total of nine islet transplants [267]. Its safety was further noted by Kim et al. who observed no deleterious effects on either islet function or gene expression [268].

The first clinical human trial implementing this imaging modality was performed recently by Saudek et al. in eight patients with T1DM [269]. No side effects related to the modality were observed. With regards to efficacy in observing pancreatic mass on MRI, they noted that the labeling period was less effective if islets were incubated with Ferucarbotran for less than 16 hours. Decrease in visualization occurred one week following transplantation, thus, correlating with the oft observed early destruction of islets. This also corresponds with a previous study in rats [270]. Thereafter, visualization remained stable for up to 24 weeks. As they concluded, while the modality allows for precise localization and quantification, an exact correlation between total number of transplanted islets and hypointense spots (as observed on MR) should not be expected due to a number of suspected factors including islet cell destruction, islets seeding together, lack of detection in counting, and random decreased contrast uptake. 

While these studies are in their early phases for clinical application, they represent important steps towards enhancing the monitoring of islet cell transplantation [269]. Recently, ferumoxide has also been introduced as a labeling agent. Though it exhibits a similar safety profile to Ferucarbotran, it exhibited inferior iron uptake by islet cells and increased hepatic clearance, thus, affording less background [271–274]. In a distinctive use of the MRI, manganese-enhanced MRI has also been used to successfully quantify *β*-cell mass in both static and dynamic conditions without manganese-associated toxicity, otherwise characterized by changes in insulin production. While still in its research phase of implementation, this may yet serve as another potential avenue for further research of islet graft monitoring [275].

In addition to MRI, bioluminescent imaging has also been a source of optimism for its potential use in posttransplant islet monitoring [276–281]. First performed by Lu et al., the group transduced isolated human and rodent islets with recombinant adenovirus or lentivirus vectors expressing a firefly luciferase gene under the control of the nonspecific cytomegalovirus promoter. The promoter is not subject to regulation by blood glucose levels so as to accurately reflect the remaining islet graft mass [276]. Luciferase, when it reacts with its substrate, D-luciferin produces a photon emission that may be detected by a cooled charge-coupled device (CCD) camera. Following implantation, they discovered that the CCD signal was proportional to the implanted islet graft mass and that the lentivirus-engineered islets could be repetitively imaged long term after transplantation [276]. In a follow-up study, Chen et al. implemented the bioluminescent imaging model to determine how a change in functional islet mass correlated with metabolic abnormalities during the course of posttransplant rejection. They found that imaging modality was very sensitive, with bioluminescent signals observed from as few as 10 islets implanted in a variety of locations. Intensity stabilization occurred within two weeks and remained so for as long as 18 months after transplant [277]. Virostko et al. and Grossman et al. also implemented the bioluminescence model with transgenic mice expressing luciferase and obtained similar results, noting that small changes in recovery of bioluminescence correlated with major changes in blood glucose control [279–281].

As mentioned earlier, in another novel technique, Witkowski et al. examined an intramuscular transplantation site and followed graft progression using positron emission tomography (PET) imaging with [11C] dihydrotetrabenazine. The site was pretreated with a biocompatible angiogenic scaffold, which was found to significantly improve engraftment versus control models. PET imaging visualized and quantified the islet mass and also correlated with the maintenance of normoglycemia by the islet graft [226].

In conclusion, imaging of islet cell mass has made significant strides in the past few years as novel areas of research develop all while new ones continue to spring up. And while such research gives enthusiasm for the potential to improve the evasive in vivo monitoring of islet survival and functionality, there is still some work required to establish a more reproducible and readily universally applicable modality to have a clinical impact.

## 10. Autotransplantation for Chronic Pancreatitis

The concept of islet autotransplantation following pancreatic resection as a surgical treatment option in patients with chronic pancreatitis first developed at the University of Minnesota (UMN) in the 1970s, when Najarian et al. indicated therapeutic value in its role to relieve the pain in this patient population [17, 282, 283]. However, the side effect of diabetes must be taken into consideration when considering it as an alternative [17]. This has an important role in considering when to revert to surgery for treatment, as delaying in patients with chronic pancreatitis may lead to progressive damage of the pancreas and a subsequent decline in islet yield [284, 285].

In a recent retrospective study, it was found that up to 80% of patients had reduced or eliminated the need for narcotics [213]. Pain relief is obtained in most patients, and health-related quality of life is significantly improved [286–291]. A UMN analysis showed that nearly 95% of adult patients had less pain following surgery [292]. Insulin independence is preserved long term in about one-third of patients, with another third having sufficient beta-cell function so that the resulting diabetes is mild and easily controlled [287]. While there is a decline in graft function over time, long-term insulin secretion remains evident and may protect against long-term diabetic complications [293].

In a case study by Illouz et al., a patient suffering from chronic pancreatitis for more than two years and abnormal glucose tolerance test underwent pancreatectomy and islet autotransplantation and remains insulin independent 5 years after transplantation with less than 1,000 IEQ/kg body weight [294]. Interestingly, the same group has shown that no significant correlation exists between the number of islets transplanted and insulin independence. However, this may be as a result of differences in the etiology of chronic pancreatitis, such as secondary to chronic alcoholism [295]. This was in contrast to the University of Minnesota series [296]. Altogether, it is clear that islet graft function and efficacy following autotransplantation are greater, even with the presence of a lower *β*-cell mass [296, 297]. 

This same procedure has also been shown to have success in the pediatric population [298–300]. At the University of Minnesota, in 18 patients surveyed under the age of 18 suffering from chronic pancreatitis who underwent pancreatectomy and islet autotransplantation, only 7 were on narcotics, and 10 were insulin independent at 1 year. They concluded that the severity of diabetes may be reduced in three-fourths of patients, with higher graft efficacy in younger patients [298]. They also have identified that in the pediatric population, as in adults, performing the procedure early in the disease course is best to preserve islet cell mass and that preoperative measurement of fasting plasma glucose is useful for predicting islet yield [301, 302]. Furthermore, any surgical procedures prior to pancreatic resection should be avoided [299, 302].

## 11. Conclusion

Islet cell transplantation for the treatment of diabetes mellitus has made remarkable strides in its evolution towards truly becoming an alternative treatment to intensive medical therapy and pancreas transplantation. However, as highlighted in this paper, while barriers are identified and advancements are made, progress remains until it may be considered a more efficacious and viable alternative to those already established. Having identified several areas that serve against islet survival in the peritransplant period, there is reason to remain optimistic that new therapies and protocols will be implemented and, thus, aid towards the gradual improvement of islet graft efficacy over the short and long course. In the end, we can hope that islet cell transplantation will serve to prevent the debilitating complications of diabetes mellitus and lead our patients to healthier lives.
